# Inhibition of Matriptase Activity Results in Decreased Intestinal Epithelial Monolayer Integrity *In Vitro*

**DOI:** 10.1371/journal.pone.0141077

**Published:** 2015-10-21

**Authors:** E. Pászti-Gere, S. McManus, N. Meggyesházi, P. Balla, P. Gálfi, T. Steinmetzer

**Affiliations:** 1 Szent István University, Faculty of Veterinary Science, Department of Pharmacology and Toxicology, Budapest, Hungary; 2 Semmelweis University, 1st Department of Pathology and Experimental Cancer Research, Budapest, Hungary; 3 Philipps University Marburg, Faculty of Pharmacy, Institute of Pharmaceutical Chemistry, Marburg, Germany; University Hospital Hamburg-Eppendorf, GERMANY

## Abstract

Barrier dysfunction in inflammatory bowel diseases implies enhanced paracellular flux and lowered transepithelial electrical resistance (TER) causing effective invasion of enteropathogens or altered intestinal absorption of toxins and drug compounds. To elucidate the role of matriptase-driven cell surface proteolysis in the maintenance of intestinal barrier function, the 3-amidinophenylalanine-derived matriptase inhibitor, MI-432 was used on porcine IPEC-J2 cell monolayer. Studies with two fluorescent probes revealed that short (2 h) treatment with MI-432 caused an altered distribution of oxidative species between intracellular and extracellular spaces in IPEC-J2 cells. This perturbation was partially compensated when administration of inhibitor continued for up to 48 h. Significant decrease in TER between apical and basolateral compartments of MI-432-treated IPEC-J2 cell monolayers proved that matriptase is one of the key effectors in the maintenance of barrier integrity. Changes in staining pattern of matriptase and in localization of the junctional protein occludin were observed suggesting that inhibition of matriptase by MI-432 can also exert an effect on paracellular gate opening via modulation of tight junctional protein assembly. This study confirms that non-tumorigenic IPEC-J2 cells can be used as an appropriate small intestinal model for the *in vitro* characterization of matriptase-related effects on intestinal epithelium. These findings demonstrate indirectly that matriptase plays a pivotal role in the development of barrier integrity; thus matriptase dysfunction can facilitate the occurence of leaky gut syndrome observed in intestinal inflammatory diseases.

## Introduction

Matriptase, a type II transmembrane serine protease encoded by the suppressor of tumorigenicity-14 (ST-14) gene, can be found in all types of epithelial tissues including normal tissue and epithelial derived cancer cells. Matriptase contributes to metastatic acitvity [1] and has been linked to various other pathological processes. It is a key initiator of cartilage destruction in osteoarthritis [2] while other studies have shown that the pathogenesis of several intestinal diseases such as Crohn's disease, ulcerative colitis and inflammatory bowel disease (IBD) is accompanied by increases in the permeability of the intestinal barrier via altered modulation of matriptase [3–4].

The intestinal epithelial barrier provides important protection against toxins, bacteria and contributes to the ion and water homeostasis and to prevention of physico-chemical stresses. Matriptase appears in abundance in the digestive tract especially in the small intestine and it shows a graded presence immunohistologically from the crypt cells to the villus with the highest matriptase activity towards the villus tip [5]. Matriptase seems to modulate the intestinal epithelial barrier function in skin and intestine and it can act as a primary serine protease in tight junction (TJ) assembly in cell surface proteolytic events [6]. A study carried out using ST-14 hypomorphic mice (100-fold less intestinal matriptase mRNA compared to controls) showed that inhibition of matripase results in a decrease in transepithelial electrical resistance and an increased paracellular permeability [3].

The paracellular pathway is regulated by apical junction complexes (AJC) which consist of apically located tight junctions, lateral adherens junctions and desmosomes [7]. Matriptase is expressed during the differentiation of the epithelium and is colocalised with E-cadherin on these AJCs. Claudin-2 is a protein present in the tight junction that is responsible for "loosening" the junction and this protein is overexpressed after inhibition of matriptase [8]. Inflammatory cytokines which are present in abundance in the aforementioned small intestinal pathologies are thought to be capable of inhibiting the expression of matriptase and therefore prevent the ability of the intestinal epithelium to recover its barrier integrity [4].

Intestinal permeability is also affected by oxidative stress caused by various reactive oxygen species (ROS). Excessive amounts of endogenously produced ROS which could not be neutralised by antioxidants lead to oxidative stress-induced gut leakage including severe cellular membrane damage and increased permeability [9]. Fluorescent probes such as 2’, 7’-dichlorodihydrofluorescein (DCFH) [10–12] and cell-impermeable Amplex red dye [13] have been widely used due to their ability to monitor redox balance in real time and to analyse the amount of a given species produced.

Our findings, in correlation with responsiveness of IPEC-J2 cells to oxidative stress and LPS-induced inflammation [14–15], suggest that this cell type can be an appropriate *in vitro* model capable of detecting redox cellular changes when jejunal epithelium is exposed to other external stimuli such as application of matriptase inhibitors. The IPEC-J2 cells form a single confluent monolayer, become polarised, and begin to express tight junction (TJ) proteins. Claudin-4 within the TJs was co-localized with occludin resulting in formation of paracellular seal [16–17]. The advantage of using this cell line over Caco-2 is that it mimicks more closely the *in vivo* gut environment [18].

Several selective matriptase inhibitors were developed including the sulfonylated 3-amidinophenylalanine derivatives [19–20] the substrate-analogue ketobenzothiazole derivatives originated from the known P4-P1 Arg-Gln-Ala-Arg substrate sequence at the autocatalytic acivation site of matriptase [21]. Another selective small molecule matriptase inhibitor, the arginal derivative CVS-3983 could suppress the growth of androgen independent prostate tumor xenografts *in vivo* [22]. Within the series of the 3-amidinophenylalanine analogues, the dibasic derivative MI-432 possessing an N-terminal dichloro-substituted biphenyl-3-sulfonyl group and a C-terminal aminoethylpiperidide moiety was one of the most potent matriptase inhibitors. Based on a modeled complex of MI-432 in matriptase it was found that the N-terminal biphenyl group of MI-432 fits into the S3/4 pocket of matriptase making a close edge to face contact to the aromatic side chain of the characteristic matriptase residue Phe99 [19].

In our work, non-tumorigenic, non-transformed porcine jejunal intestinal epithelial IPEC-J2 cells cultured on membrane insert were treated with the 3-amidinophenylalanine-derived matriptase inhibitor, MI-432. The aim of the study was to see how matriptase inhibition affects epithelial barrier integrity based on short and long-term monitoring of transepithelial electical resistance (TER) of cell monolayer. Moreover, we have determined the influence of a reduced matriptase activity and subsequent alterations in barrier function on the redox status by quantitative fluorescence measurements of the extracellular and intracellular ROS production. The role and localization of the TJ proteins, occludin and claudin-4 in IPEC-J2 cells were also observed to see if a correlation between decrease in TER and changes in tight junctional protein distribution exists.

## Materials and Methods

### Cell lines and culture conditions

The IPEC-J2 cell line (derived from jejunal epithelia of a neonatal piglet) was kindly provided by Dr. Jody Gookin and Dr. Stephen Stauffer, Department of Clinical Sciences, College of Veterinary Medicine, North Carolina State University, Raleigh, NC, USA and it was used between passage 50 and 75. IPEC-J2 cells were seeded at a density of 1.5x10^5^ per well on six-well plates with Transwell polyester membrane inserts (pore size 0.4 μm, Sigma-Aldrich, St. Louis, MO) coated with rat tail collagen (Sigma-Aldrich, St. Louis, MO) in a 1.5 ml apical and 2.6 ml basolateral volume. The surface area of the membrane insert was 4.67 cm^2^. Cells were maintained in complete medium containing a 1:1 mixture of Dulbecco's Modified Eagle's Medium and Ham's F-12 Nutrient Mixture (DMEM/F12) supplemented with 5% FBS, 5 μg/ml insulin, 5 μg/ml transferrin, 5 ng/ml selenium, 5 ng/ml epidermal growth factor and 1% penicillin-streptomycin (all from Fisher Scientific, USA). Cell cultures were tested by PCR and were found to be free of mycoplasma contamination. Cells were allowed to adhere for 24 h before being washed and re-fed every other day until confluence. They were grown at 37°C in a humidified atmosphere of 5% CO_2_.

### Exposure of IPEC-J2 cells to selective matriptase inhibitor

Before treatment, IPEC-J2 cells were washed twice with plain medium. The solutions of inhibitor MI-432 in phenol red free DMEM at different concentrations (10, 25 and 50 μM) were prepared freshly prior to each experiment from 10 mM stock solutions, which were stored at -20°C. MI-432 was added apically for different time intervals (for 2 h, 24 h and 48 h). After incubation, the cells were washed twice with plain medium before being subjected to the subsequent procedures. TERs of confluent IPEC-J2 cells (~10 d) used for the studies reached usually ~4000–4500 Ω× cm^2^ after growing further 2 days (differentiated cells).

### Neutral Red uptake assay for cell viability

For testing of the influence of inhibitor MI-432 on the viability of enterocytes IPEC-J2 cells were seeded in a 96-well plate and incubated with 50 μM MI-432 for 24 and 48 h, respectively. The control cells were incubated only with phenol red free DMEM. After removal of the medium and washing, 45 mg/l Neutral red solution (Sigma-Aldrich, St. Louis, MO) was added to the IPEC-J2 cells in plain phenol red free medium for 2 h. After washing the IPEC-J2 cells, destaining solution (ethanol/demineralised water/glacial acetic acid, 7.5/7.4/0.15, v/v/v) was applied for 10 min. Viability of IPEC-J2 cells was measured 24 and 48 h after treatment with inhibitor MI-432 at 540 nm by Neutral Red uptake assay using a EZ Read Biochrom 400 microplate reader [23].

### Transepithelial electrical resistance

IPEC-J2 cells were plated to confluence in 6-well polyester membrane inserts and were allowed to form confluent monolayers. The cells were washed three-fold and incubated with inhibitor MI-432 (10, 25 and 50 μM) for different time intervals (2, 24 and 48 h) in phenol red free DMEM. The MI-432 solution was then removed by washing the cells three times with phenol red free DMEM. TER measurements of cell layers were performed prior to and immediately after MI-432 administration using an EVOM Epithelial Tissue Volt/Ohmmeter (World Precision Instruments, Berlin, Germany). The average baseline electrical resistance of the polyester membrane insert without IPEC-J2 cell layer was 113 Ω, which was substracted from actual TER data.

### Intracellular DCFH fluorogenic probe

Measurement of perturbances in intracellular redox state of IPEC-J2 cells was carried out using 2’, 7’-dichlorodihydrofluorescein-diacetate (DCFH-DA) dye (Sigma-Aldrich, St. Louis, MO). In this form, the probe can penetrate the cell membrane before being deacylated by cellular esterases to another non-fluorescent form, DCFH. DCFH is then oxidised to the highly fluorescent form dichlorofluorescein (DCF) by the intracellular ROS. IPEC-J2 cells were treated with matriptase inhibitor MI-432 at 10, 25 and 50 μM for 2, 24 and 48 h in phenol red free DMEM. A working solution of 10 μM DCFH-DA was added and incubated for 30 min after which a quantitive analysis of the intracellular ROS activity was carried out using the Victor X2 2030 fluorometer (λ_ex_ = 480 nm, λ_em_ = 530 nm).

### Extracellular H_2_O_2_ measurement by the Amplex red method

Fluorescent ROS measurement of cell supernatant was based on the detection of H_2_O_2_ using the Amplex Red Hydrogen Peroxide Assay Kit (Invitrogen, Molecular Probes). In the presence of horseradish peroxidase (HRP), Amplex Red reacts with H_2_O_2_ in a 1:1 stoichiometry producing a highly fluorescent resorufin [24]. IPEC-J2 cells were treated with matriptase inhibitor MI-432 at 10, 25 and 50 μM for 2, 24 and 48 h in phenol red free DMEM and the H_2_O_2_ concentrations in the medium were determined using a working solution of 100 μM Amplex Red and 0.2 U/ml HRP. After 30 min incubation with the dye at room temperature the quantitative H_2_O_2_ contents was measured using a Victor X2 2030 fluorometer (λ_ex_ = 560 nm, λ_em_ = 590 nm).

### Protein extraction

For protein extraction the IPEC-J2 cells were washed with PBS and harvested with cell scraper after adding 250 μl extraction buffer containing 20 mM Tris pH 7.4 2 mM EDTA, 150 mM NaCl, 1% Triton X-100 supplemented with 10 μl/ml phosphatase inhibitors (Sigma-Aldrich, Steinheim, Germany) and 5 μl/ml proteinase inhibitors (Sigma-Aldrich, Steinheim, Germany). For phosphatase treatments of protein extract, extraction buffer was used containing 20 mM Tris pH 7.4, 150 mM NaCl, 1% Triton X-100. The pellet was collected in 1.5 ml Eppendorf tube and lysed for 30 minutes on ice. Lysates were then centrifuged at 12 000 rpm for 15 min at 4°C. The extracts were mixed with 5x Laemmli sample buffer containing 5% 2-mercaptoethanol (BioRad Laboratories, Philadelphia, PA, USA) and heated to 95°C for 5 min. Protein concentration in the supernatant was determined by the Bradford assay (BioRad Laboratories, Philadelphia, PA, USA).

### Western blot

Analysis of matriptase in cell extract was performed by Western blotting. Equal amounts of protein (20 μg) were loaded and run on a 10% sodium dodecyl sulfate polyacrylamide gel electrophoresis (SDS-PAGE) at 180 V for 1 h. After running the membrane was transferred onto Immobilion-P nitrocellulose membrane (Millipore, Billerica, Germany) at constant 75 mA at 4°C overnight. After blotting, the membrane was stained with ponceau S red (BioRad Laboratories, Philadelphia, PA, USA) to visualize the transferred proteins. Nonspecific binding of the antibody was blocked by incubation with 5% non-fat milk (BioRad Laboratories, Philadelphia, PA, USA) dissolved in 1x 0.1 M Tris-buffered saline (TBS) containing 0,05% Tween 20 pH 7.4 (TBST) for 60 min. The membrane was washed 5 times for 5 min with 1x TBST, and incubated overnight at 4°C with rabbit polyclonal anti-human anti-ST-14 N terminal (1:500, Sigma-Aldrich, St. Louis, MO) antibody diluted in 1xTBST containing 3% non-fat milk. Next day the membrane was washed 5 times for 5 min with TBST and incubated with horseradish peroxidase (HRP) conjugated goat anti-rabbit secondary antibody (1:1000, Cell Signaling) diluted in 1xTBST containing 1% non-fat milk for 60 min at room temperature. For loading control rabbit anti-human β-actin (1:2000, Cell Signaling) antibody was used for 60 minutes. Detection was performed by Super Signal West Pico ECL reagent for 10 min (Pierce Biotechnology Inc., Rockford, IL, USA). The molecular mass of specific bands was determined comparing to the Precision Plus Protein Standard (BioRad Laboratories, Philadelphia, PA, USA) applied on the same gels. Densitometric analysis of the blots was done with Kodak Molecular Imaging Software 4.1 in a Kodak Image Station 4000 MM (Kodak, Rochester, NY, USA).

### Investigation of matriptase, claudin-4 and occludin distribution via immunfluorescent staining

Inserts were fixed in methanol for 5 min followed by bovine serum albumin (BSA (5%, Sigma Aldrich, St Luis, MO) protein block for 20 min. Sections were incubated for 1 h in a humid chamber at room temperature with anti-ST-14 N terminal rabbit polyclonal primary antibody (1:200, Sigma-Aldrich, St. Louis, MO), anti-claudin-4 (1:200, Thermo Scientific Fremont, CA, USA) or anti-occludin polyclonal primary antibody (1:200, Sigma-Aldrich, St. Louis, MO), the antibodies were previously diluted in 5% BSA solution. For secondary antibody Alexa546 (orange-red) anti-rabbit Ig-s 1:200 diluted in PBS was used for 1 h. Sialic acid residues in cell membrane were stained with wheat germ agglutinin (WGA) (1:200 diluted in PBS, WGA Alexa Fluor 488, Invitrogen-Molecular Probes) for 10 min and cell nuclei were stained in blue using 4’,6-diamidino-2-phenylindole (DAPI) (1:500 diluted in PBS, Invitrogen-Molecular Probes) for additional 10 min. Between incubations the slides were washed in phosphate-buffered saline buffer (PBS) for 3x2 min. Membranes were attached on glass slides using fluorescent mounting medium (DAKO, Glostrup, Denmark). The samples were analyzed using a Nikon Eclipse E600 epifluorescent microscope with LUCIA™ Citogenetics 2.5 software.

### Statistical analysis

For statistical evaluation R 2.11.1 software package (2010) was applied. Differences between means were evaluated by one-way analysis of variance (one-way ANOVA) with post-hoc Tukey test, where data were of normal distribution and homogeneity of variances was confirmed. Differences were considered significant if the p value was <0.05 marked with * (**p< 0.01, ***p< 0.001)

## Results

### Cytotoxicity study with Neutral red method

The cytotoxic effect of inhibitor MI-432 on IPEC-J2 was investigated using the Neutral red method. It was found that treatment with inhibitor MI-432 at the highest dose of 50 μM used in this study over a period 48 h did not affect IPEC-J2 cell viability compared to that of untreated cells (S1 Fig).

### Expression of matriptase in IPEC-J2 cells

We first studied the expression of matriptase in lysates of IPEC-J2 cells. Matriptase was detected as single bands of 95 kDa using Western blotting (S2 Fig). The expression of matriptase did not change even when IPEC-J2 cells were treated with inhibitor MI-432 at 50 μM for 48 h. The houskeeping protein β-actin appeared as 42 kDa band on the blot.

### MI-432-induced alterations in transepithelial electrical resistance of IPEC-J2 cell monolayer

**Fig 1:**
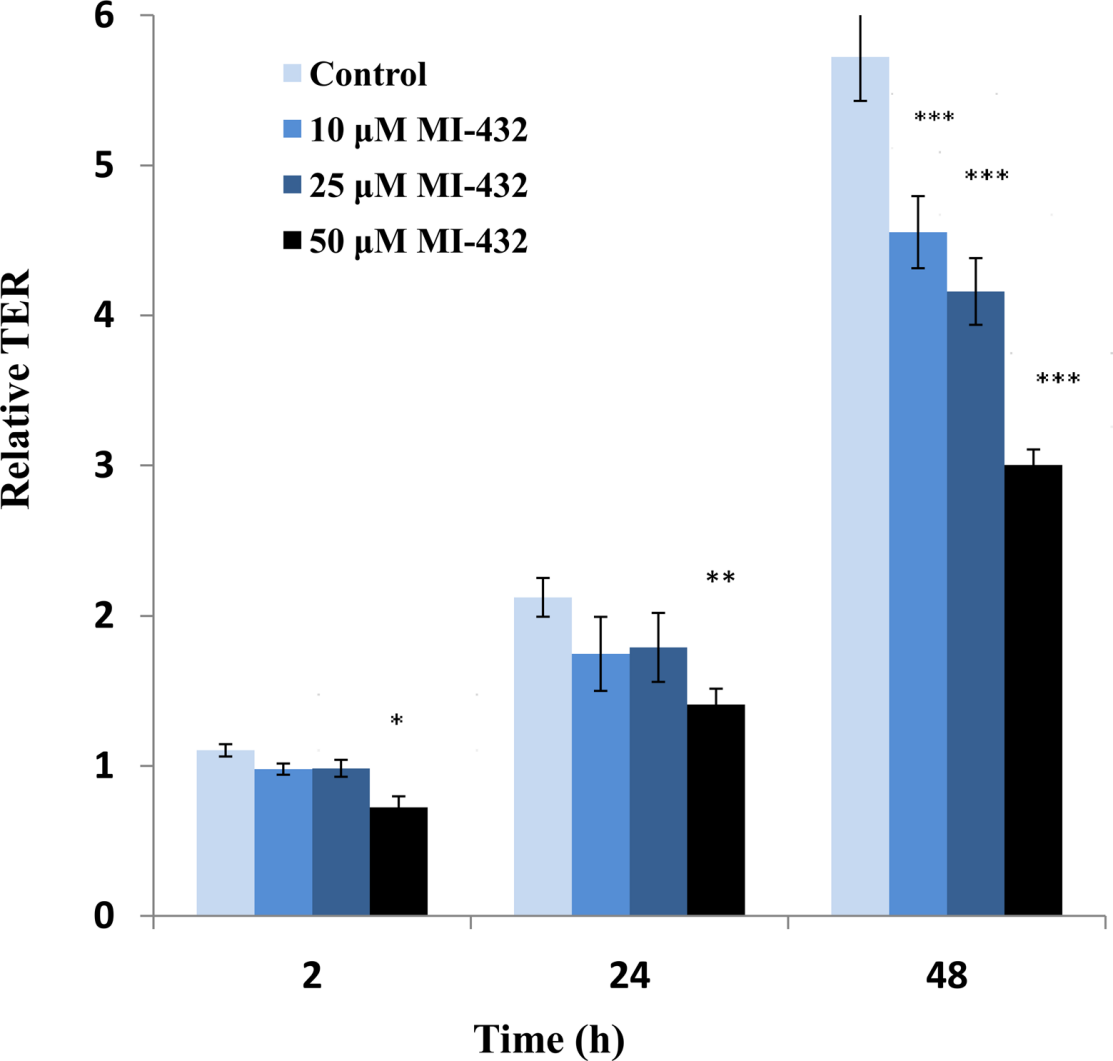
Relative TER (actual TER values divided by initial TERs) of both the control and the inhibited cell cultures (at 10, 25 and 50 μM of inhibitor MI-432). At 2 hours (*p < 0.05) and 24 hours (**p < 0.01) significant decreases in TER can be seen only with the 50 μM concentration while at 48 hours, for all concentrations of MI-432 (***p < 0.001) there are significant decreases in the TERs when compared to control cells.

TER was measured on the control and inhibitor treated monolayers (Fig 1). The results shown represent the relative TERs between actual and initial values for the various timeframes. The initial TER values were quite low (approx 600–800 Ω× cm^2^) indicating that the cell culture had not fully differentiated. At 48 h TER has reached a plateau of ~ 4000–4500 Ω× cm^2^ indicating that tight junctions have been established between the highly polarised adjacent IPEC-J2 cells. However, it was found that even prior to reaching complete differentiation there is a negative effect on the TER in the cultures treated with MI-432. After 2 h and 24 h incubation time it can be seen that TER is decreased significantly by addition of 50 μM MI-432 (*p< 0.05 and **p< 0.01, respectively). A concentration-dependent decrease in TER was also found when IPEC-J2 cells were treated with MI-432 for longer periods of time (48 h). For instance, a significant reduction in TERs was observed when the cell monolayer was exposed to the lowest 10 μM concentration of inhibitor MI-432 (in case of all three concentrations of the inhibitor ***p < 0.001).

### Changes in ROS level in IPEC-J2 cells exposed to inhibitor MI-432

**Fig 2:**
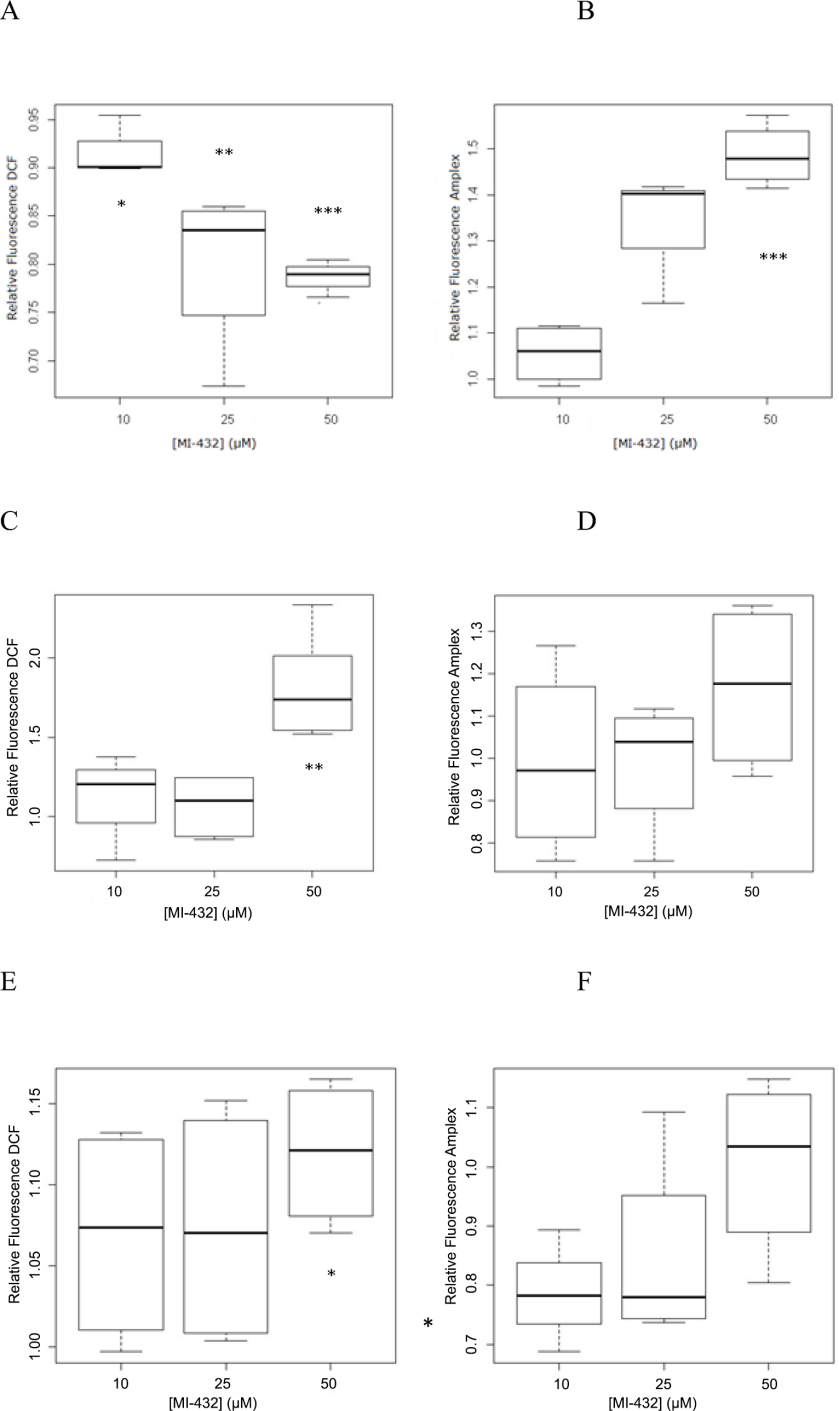
Quantitative analyses of ROS levels in IPEC-J2 cells incubated for 2 hours (2A, 2B), 24 hours (2C, 2D) and 48 hours (2E, 2F) with inhibitor MI-432 at different concentrations (10, 25, 50 μM). Fig 2 A, C, E represent intracellular ROS levels determined by relative fluorescence intensities ± SDs using DCF. Fig 2 B, D, F represent extracellular H_2_O_2_ levels measured by relative fluorescence intensities ± SDs using Amplex Red. * Indicates significant differences between the fluorescence values of the treated and control cells (*p < 0.05, **p< 0.01, ***p< 0.001, n = 3).

After a 2 hour incubation time with inhibitor MI-432 at 10 μM, 25 μM, and 50 μM fluorescence intensities of both the control and treated samples were measured. It was found that there is an acute decrease in the intracellular ROS activity with the decrease being more pronounced as the concentration of the inhibitor is increased (Fig 2A). All three inhibitor concentrations cause a significant change (p = 0.0194 for 10 μM, p = 0.0077 for 25 μM, p = 0.0001 for 50 μM) which may be representative of putative outflow of hydrogen peroxide from the cells. For this reason it was important to compare these findings with the corresponding extracellular oxidative stress using Amplex Red. The results showed that there was an increase in the extracellular ROS amounts (Fig 2B) causing a significant elevation in fluorescence when inhibitor MI-432 was applied at a concentration of 50 μM (p = 0.0007). After a 24 hour incubation time with MI-432 at 10 μM, 25 μM, 50 μM, a significant increase of intracellular ROS can be seen with the 50 μM concentration of inhibitor MI-432 (p = 0.0014) while no significant changes were found in the extracellular compartment for any concentrations of the inhibitor (Fig 2C and 2D). This measurement shows an almost two fold increase in the intracellular ROS activity compared to the control group, which indicates that 24 hour inhibition of matriptase causes marked disturbances in the ROS production inside the cells. The extracellular fluorescence was, however, not significantly elevated. After a 48 hour incubation time with the inhibitor MI-432 at 10 μM, 25 μM and 50 μM fluorescence values of DCF were determined in control and in treated IPEC-J2 cells. The results show that there were not any perturbations in redox balance in IPEC-J2 cells for the 10 μM and 25 μM concentrations of the inhibitor and less significant relative increase (*p = 0.014) after treatment with 50 μM inhibitor than was present after 24 h. In the extracellular compartment, there were not any significant increases in ROS levels (Fig 2E and 2F).

### MI-432-induced changes in tight junctional protein assembly

**Fig 3:**
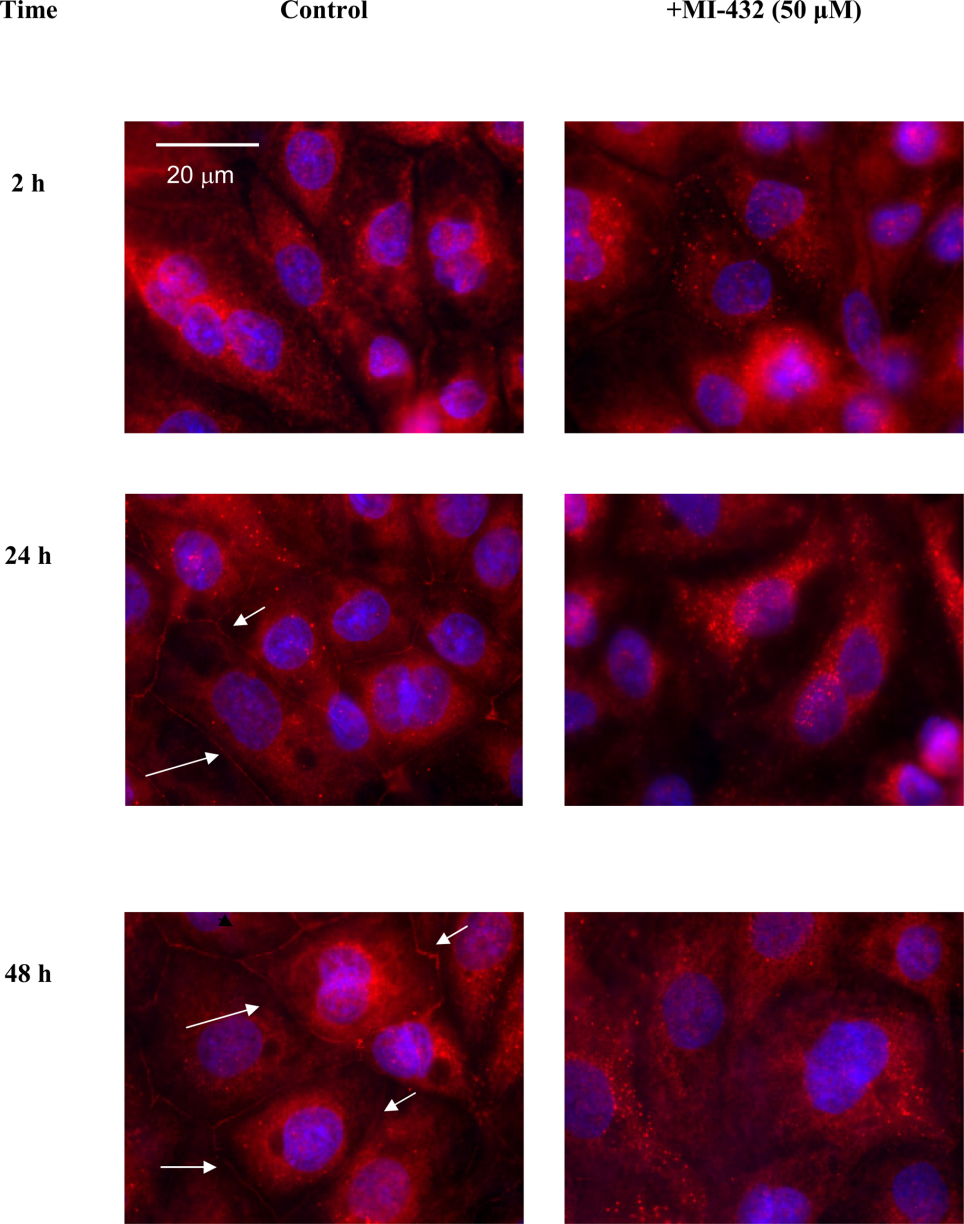
Immunofluorescent staining of occludin (in red) in control and in inhibitor MI-432-treated IPEC-J2 cells after different treatment times. The cells were not completely differentiated when the inhibitor administration (50 μM) was started. Occludin can be seen in red and cell nuclei were stained with DAPI in blue. MI-432-induced matriptase suppression resulted in occludin loss in TJ strands. White arrows indicate the characteristic membranous pattern of occludin presence in the control panel.

Localizaton of occludin in TJ assembly was studied in untreated control and in inhibitor-treated not fully differentiated IPEC-J2 cells using immunfluorescence staining. The cells were investigated 2 h, 24 h, 48 h after MI-432 treatment. In controls, occludins localized at the cell membrane of IPEC-J2 cells as cell differentiation proceeds and they were mainly present in tight junction in polarized monolayers. In contrast, in cells exposed to inhibitor MI-432 at 50 μM concentration occludin remained in the cytoplasm (Fig 3). The localization pattern of occludin significantly changed when inhibitor MI-432 was continously administered and the loss of membranous occludin from TJ can be an explanation to TER changes observed even when the 10 μM MI-432 was administered to the IPEC-J2 cells for 48 h.

**Fig 4:**
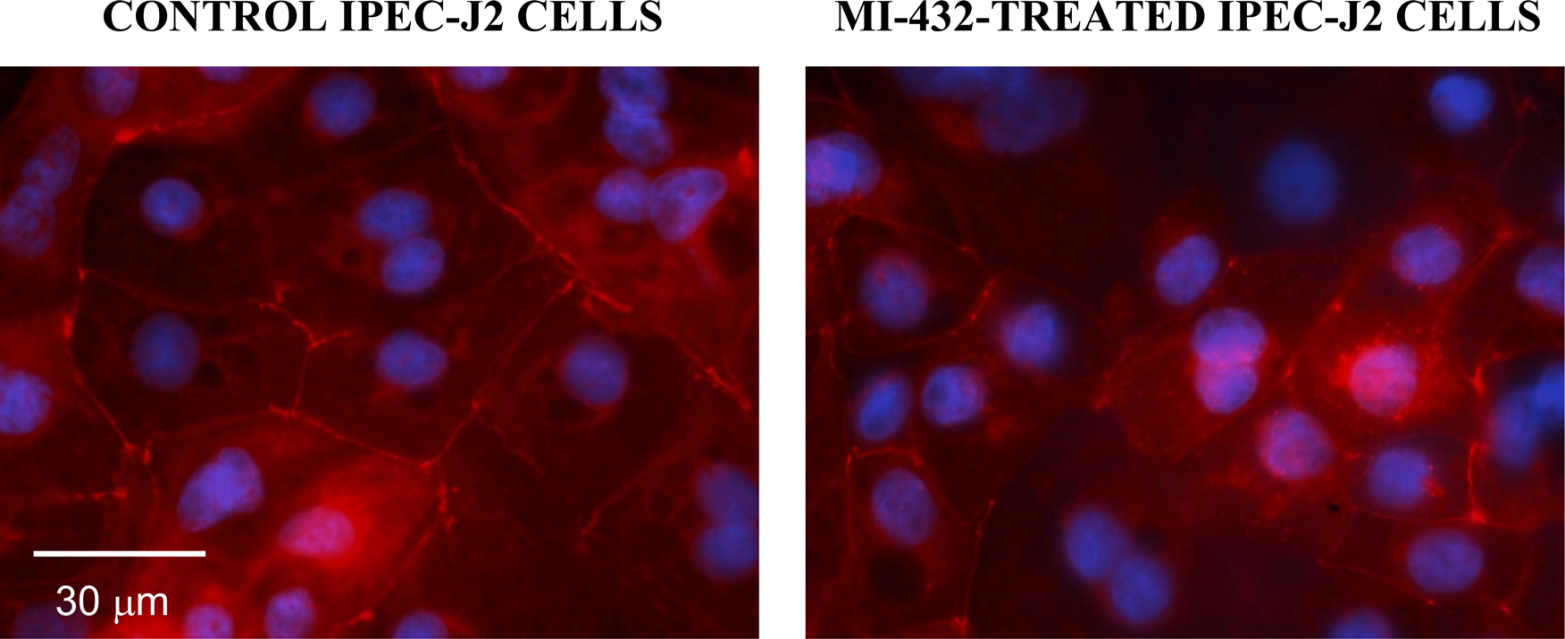
Immunofluorescent staining of claudin-4 in control and MI-432-treated IPEC-J2 cells grown on Transwell polyester membrane insert. Cell monolayers were incubated with plain DMEM (control) or MI-432 (treated) for 48 hours, and then cells were labelled for claudin-4 and cell nuclei were stained with DAPI in blue. IPEC-J2 cells showed homogenous, intense membranous claudin-4 positivity in the absence and in the presence of MI-432.

In order to estimate the impact of MI-432 treatment on another TJ protein, subcellular localization of claudin-4 was also investigated by immunofluorescence. Results showed that in contrast to occludin loss from the tight junction strands the distribution of the claudin-4 was unaltered after treatment of cell monolayers with 50 μM MI-432 (Fig 4).

### Subcellular localization of matriptase in IPEC-J2 cells

**Fig 5:**
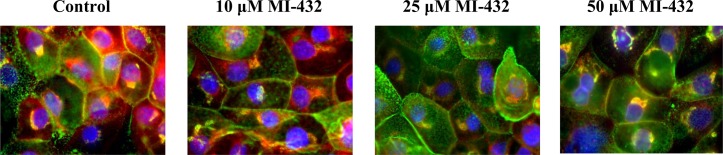
Immunostaining of matriptase expression in IPEC-J2 cells cultured on polyester membrane inserts treated with inhibitor MI-432 for 48 hours. Cell nuclei are stained blue (DAPI), cell membranes are labelled with wheat germ agglutinin (Alexa 488, green). In control samples matriptase (Alexa 564 red) is colocalized with wheat germ agglutinin. 48 h long treatment of MI-432 at higher concentrations (at 25 and 50 μM) induced significant fluorescent signal loss of matriptase. Matriptase occurence was mainly observed around synthesis sites in MI-432-treated IPEC-J2 cells. 600 × magnification.

IPEC-J2 cells were exposed to inhibitor MI-432 for 48 h and cellular distribution of matriptase was analyzed by epifluorescent miscroscopy. The matriptase can be found on the cell surface with more intense staining pattern at cell-cell contacts and within the enterocytes where the staining was rather diffuse. Immunfluorescence analysis revealed that in control samples wheat germ agglutinin (WGA) was co-localized with matriptase confirming the cell membranous positivity and perinuclear presence of matriptase under physiological conditions. With increasing concentration of MI-432, remarkable changes in distribution pattern of matriptase was detected in IPEC-J2 cells. By the addition of inhibitor MI-432 at 10 μM, matriptase was still present in the cell membrane and in the cytoplasm, however, when MI-432 concentration was increased at 25 and 50 μM only smaller amounts of matriptase could be detected (Fig 5), presumably at the site of the synthesis with a visible decrease in the membrane presence of matriptase.

## Discussion

The intestinal epithelium is of key importance for the regulated absorption of nutrients, protection against invasive pathogens or toxins and in the facilitation of crosstalks between the GI tract and immune system. The epithelium of the human digestive tract, especially that of small intestine, shows strong matriptase immunoreactivity along the crypt-villous axis. It was found that villous enterocytes possess progressively stronger expression of matriptase compared to reduced levels found in crypt cells [5]. The question why the intestine is enriched with the trypsin-like transmembrane protease matriptase and what is their physiological role in the development and maintenance of intestinal epithelial integrity has not been fully answered yet.

A few studies searching for a relationship between barrier homeostasis and matriptase activity have been conducted on human intestinal cell lines such as Caco-2 and T84 derived from a cancerous colon [3–4, 6]. It was demonstrated in previous work that a significant decrease in TER and parallel elevation in FD4 were detected, when Caco-2 cells were treated with the broad-spectrum trypsin-like serine protease inhibitor AEBSF (Pefabloc SC) or with the more selective matriptase inhibitor CVS-3983. This indicated an enhanced paracellular permeability of the cell monolayer after matriptase inhibition. Based on *ex vivo* experiments deficiency of matriptase in *St14* hypomorphic mice caused enhanced claudin-2 expression and it delayed the recovery of barrier integrity, when intestinal mucosa was injured during dextran sodium sulfate-induced colitis [4]. In accordance, embryonic or postnatal ablation of *St14* in epithelial tissues resulted in increased permeability and mislocation of TJ proteins [25]. Barrier development was facilitated by addition of recombinant matriptase in cancerous cells thus raising the question whether matriptase can also stimulate barrier formation in case of non-tumorigenic epithelial cell lines.

It was recently reported that serine protease activity is a prerequisite for healthy gut function based on the results with non-tumorigenic non-transformed cell lines. The advantage of intestinal epithelial cell (IEC) lines not derived from tumors over cancerous Caco-2 cells is its closer resemblance to behavioural and physiological situations *in vivo*. Apical treatment of a canine epithelial cell line, SCBN with trypsin or matriptase induced fast and prolonged increase in TER and decrease in paracellular permeability detected by FD4 flux. Occludin knockdown using siRNA could reduce the ability of trypsin and matriptase to induce an increase in TER and decrease in paracellular permeability in cell monolayers preexposed to IFN-γ and TNF-α [26–27]. In our studies we used porcine IPEC-J2 cells originally isolated from intestinal tissue of an unsuckled piglet. IPEC-J2 can better mimic human intestinal structure and function compared to cancer cell lines, and provide an improved *in vitro* model for investigation of close interactions between human gut epithelium and matriptase inhibition versus rodent animal models.

We had previously published that FD4 flux was elevated in IPEC-J2 cell monolayers treated with MI-432 indicating enhanced paracellular permeability by blocking the proteolytic activity of matriptase. The average fluorescence intensity was increased by 94% in 4 h, by 107% in 24 h and by 104% in 48 h in IPEC-J2 cells exposed to MI-432 at 50 μM compared to control fluorescence values measured simultaneously with the treated samples [28].

In this study IPEC-J2 cells showed intense membranous claudin-4 and occludin positivity confirming the development of differentiated phenotype. After treatment with MI-432 at 50 μM for 48 hours the cellular distribution and intensity of claudin-4 remained unaltered suggesting that matriptase inhibition had no impact on this tight junctional protein. Here we found that MI-432 evoked TER reduction between apical and basolateral compartments of cell monolayer in concentration and time -dependent manner. It was found that in the presence of inhibitor MI-432 for longer period of time up to 48 h occludin was not exclusively present in TJ assembly in IPEC-J2 cells. Thereby, it can be assumed that changes in TER induced by MI-432 treatment can at least partially correlated with relative occludin absence from TJ strands.

It was also reported that occludin knockdown resulted in selective increase in junctional claudin-2 expression in addition to affecting flux rates of macromolecules such as dextran [29]. It would be interesting to see how MI-432 addition can influence the claudin-2 abundance in TJs in IPEC-J2 cells, however, immunofluorescent staining revealed that claudin-2 fluorescent signals were significantly lower compared to those of other proteins of TJ assembly such as claudin-1, claudin-4 and claudin-7 in this cell line [30].

Previously it was observed that hydrogen peroxide administration could lead to a weakened IPEC-J2 monolayer function [9]. To see if suppression of matriptase activity could cause disruption of epithelial barrier integrity shown by TER drop after MI-432 treatment via induction of oxidative stress, ROS production was monitored quantitatively. DCF assay after 2 h treatment showed disturbances in the redox state of IPEC-J2 cells exposed to matriptase inhibition in a dose-dependent manner, and at the same time ROS concentrations in the extracellular space increased compared to untreated controls. It cannot be ruled out that changes in membrane integrity could lead to a redistribution of ROS when calcium homeostasis was still maintained. Comparing 48 h data with the shorter incubation it can be assumed that after the initial significant increase after 2 hours, there was compensation after 24 h and this compensation was maintained over 48 h.

**Fig 6:**
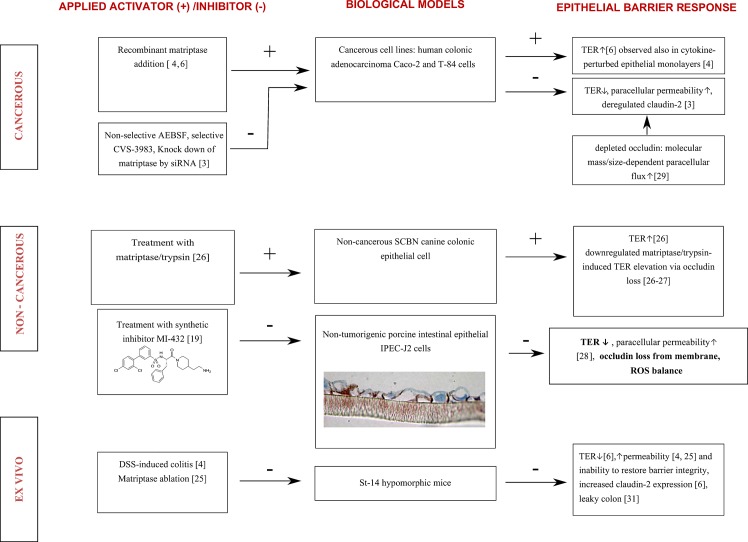
Summary of *in vitro* and *ex vivo* findings [31] after externally induced changes in matriptase-activities in the maintenance of intestinal epithelial integrity. TER stands for transepithelial electrical resistance, ST-14 means suppression of tumorigenicity-14.–indicates inhibition and + is for activation of matriptase-involved proteolytic pathways. Recent findings confirm that physiological barrier function of intestinal epithelium including transepithelial electrical resistance and paracellular efflux regulated by TJ protein assembly requires matriptase activity.

This phenomenon might refer to the fact that paracellular permeability of a cell monolayer is tightly controlled via matriptase inhibition, however, prominent ROS release can be observed mainly after acute administration of MI-432. In parallel, extracellular H_2_O_2_ tends to follow the intracellular oxidative events and the tipped equilibrium of ROS between two sides of the cell membrane was restored again. Fig 6 summarizes our findings in context with the results available from published reports including the molecular mechanisms behind the crosstalk between intestinal epithelium and matriptase in different *in vitro* and *ex vivo* systems.

In conclusion, this is the first study proving expression of matriptase in porcine non-tumorigenic IPEC-J2 cell line. To establish the relationship between the role of the proteolytic activity of matriptase and the maintenance of epithelial barrier integrity, the potent 3-amidinophenylalanine-derived inhibitor of matriptase, MI-432 was used. The MI-432-triggered cell response profile was evaluated via measurements of TER change in concentration and time-reponse manner as an indicator of intestinal epithelial barrier function. It was also concluded that acute administration of inhibitor MI-432 provoked elevated H_2_O_2_ levels detected extracellularly, which can be a result of ROS leakage from the intracellular production pool due to some kind of membrane barrier dysfunction. As for a longer timeframe the concentration of extracellularly detected peroxide was not different in control and MI-432-treated IPEC-J2 cells. Alterations in matriptase staining profile and occludin loss from TJ protein assembly in IPEC-J2 cells induced by treatment with inhibitor MI-432 were also observed.

## Supporting Information

S1 Fig Cell viability study. IPEC-J2 cells were exposed to inhibitor MI-432 at 50 μM for 24 and 48 hours. There were no significant differences between inhibitor- treated and control cells (n = 5, p< 0.05).
(DOCX)

S2 Fig Expression of matriptase in IPEC-J2 cells. IPEC-J2 cells were untreated or exposed to MI-432 for 48 hours at 50 μM in phenol red free DMEM at pH = 7.4 at 37°C. Cell lysates were prepared and analysed by immunoblotting using polyclonal ST-14 N-terminal Ab. The matriptase present in IPEC-J2 cells appeared as a 95 kDa band on the blot, whereas β-actin (42 kDa) was used as reference housekeeping protein on the same blot. Nearly identical blots were obtained in two additional parallel experiments.
(DOCX)
