# Supplementary material for: Inhibition of Matriptase Activity Results in Decreased Intestinal Epithelial Monolayer Integrity In Vitro
Source: PLoS One. 2015 Oct 21;10(10):e0141077. doi: 10.1371/journal.pone.0141077 (PMC4619522; doi:10.1371/journal.pone.0141077)

**S1 Fig. Cell viability study.** IPEC-J2 cells were exposed to inhibitor MI-432 at 50 μM for 24 and 48 hours. There were no significant differences between inhibitor- treated and control cells (n=5, p< 0.05).


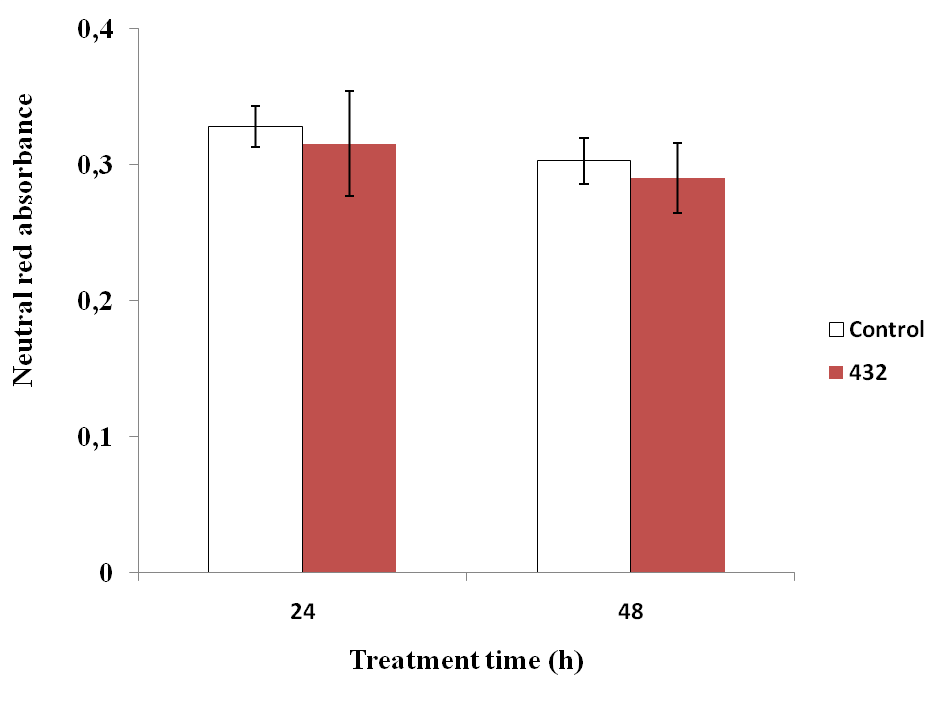

Supplement: S1 Fig — IPEC-J2 cells were exposed to inhibitor MI-432 at 50 μM for 24 and 48 hours. There were no significant differences between inhibitor- treated and control cells (n = 5, p< 0.05). (DOCX) [file pone.0141077.s001.docx]
