# Supplementary material for: Inhibition of Matriptase Activity Results in Decreased Intestinal Epithelial Monolayer Integrity In Vitro
Source: PLoS One. 2015 Oct 21;10(10):e0141077. doi: 10.1371/journal.pone.0141077 (PMC4619522; doi:10.1371/journal.pone.0141077)
Supplement: S2 Fig — IPEC-J2 cells were untreated or exposed to MI-432 for 48 hours at 50 μM in phenol red free DMEM at pH = 7.4 at 37°C. Cell lysates were prepared and analysed by immunoblotting using polyclonal ST-14 N-terminal Ab. The matriptase present in IPEC-J2 cells appeared as a 95 kDa band on the blot, whereas β-actin (42 kDa) was used as reference housekeeping protein on the same blot. Nearly identical blots were obtained in two additional parallel experiments. (DOCX) [file pone.0141077.s002.docx]

**S2 Fig. Expression of matriptase in IPEC-J2 cells.** IPEC-J2 cells were untreated or exposed to MI-432 for 48 hours at 50 μM in phenol red free DMEM at pH=7.4 at 37°C. Cell lysates were prepared and analysed by immunoblotting using polyclonal ST-14 N-terminal Ab. The matriptase present in IPEC-J2 cells appeared as a 95 kDa band on the blot, whereas β-actin (42 kDa) was used as reference housekeeping protein on the same blot. Nearly identical blots were obtained in two additional parallel experiments.
